# SCORE2 and SCORE2-OP Assessment in the Predicting of Cardiovascular Diseases and AF Recurrence in Hypertensive AF Patients Who Underwent Catheter Ablation

**DOI:** 10.3390/jcm15010290

**Published:** 2025-12-30

**Authors:** Gülhan Yüksel, Mustafa Lütfullah Ardıç, Hilmi Erdem Sumbul, Mevlut Koc

**Affiliations:** 1 Department of Cardiology, University of Health Sciences, Adana Health Practice and Research Center, Adana 01230, Turkey; drmustafaardic@gmail.com (M.L.A.); mevlutkoc78@yahoo.com (M.K.); 2 Department of Internal Medicine, University of Health Sciences, Adana Health Practice and Research Center, Adana 01230, Turkey; erdemsumbul@gmail.com

**Keywords:** atrial fibrillation, hypertension, catheter ablation, recurrence, SCORE2 and SCORE2-OP

## Abstract

**Background/aims**: Hypertension (HT) is a modifiable risk factor for the development of cardiovascular disease (CVD) in patients with atrial fibrillation (AF). There are no data on the use of the SCORE2 and SCORE2-OP risk scores used in patients with AF. In our study, we aimed to determine the effect of SCORE2 and SCORE2-OP risk scores on AF recurrence and CVD development after catheter ablation (CA) in AF patients with HT. **Methods**: This retrospective cohort study included 266 patients (144 men, 122 women, 57.1 ± 11 years) who underwent CA with a diagnosis of paroxysmal AF. Patients were grouped as <5%-5% to <10%-≥10% (Group I-II-III, respectively) according to CVD risk in SCORE2 and SCORE2-OP. The primary endpoint was adverse CVD. The secondary endpoint was AF recurrence. **Results**: The frequency of CVD and AF recurrence in groups I-II-III was 0(0%)-8(8%)-14(15%) and 9(13%)-17(16%)-23(25%), respectively (*p* = 0.001 and *p* = 0.035). Age, systolic blood pressure, presence of CVD risk ≥ 10%, CHA_2_DS_2_-VA, BUN, and uric acid were found to be higher in those with CVD. In logistic regression analysis, the presence of CVD risk ≥ 10%, CHA_2_DS_2_-VA and uric acid were found to independently predict the development of CVD (OR = 3.960, 95%CI-1.098–8.508, *p* = 0.019, OR = 3.257, 95%CI-1.067–7.318, *p* = 0.015 and OR = 1.967, 95%CI-1.359–2.873, *p* = 0.001). Triglycerides, CVD risk of ≥10%, and left atrial end-diastolic diameter (LAd) were found to be higher in those with AF recurrence. In regression analysis, the presence of CVD risk ≥ 10% and LAd were found to independently determine the development of AF recurrence (OR = 2.448, 95%CI-0.993–1.023, *p* = 0.005, OR = 1.217, 95%CI-1.124–1.319). **Conclusions**: SCORE2 and SCORE2-OP algorithms performed before the procedure in isolated HT patients undergoing ablation for paroxysmal AF can be used to predict AF recurrence and CVD development. The SCORE2 and SCORE2-OP algorithms have been evaluated exclusively in patients with isolated hypertension; therefore, further studies are needed to determine their applicability in the broader atrial fibrillation population.

## 1. Introduction

Hypertension (HT) is the most important modifiable risk factor for adverse cardiovascular (CV) outcomes [1]. Uncontrolled HT is associated with hypertension-mediated organ damage (HMOD) [1]. Cardiac involvement of HMOD may persist subclinically for long periods of time: (i) atrial fibrillation (AF), (ii) left atrial (LA) and left ventricular (LV) dilatation, (iii) impaired LV systolic and diastolic function and heart failure (HF), (iv) LV hypertrophy, (v) obstructive and non-obstructive coronary artery disease (CAD), (vi) myocardial infarction, and (vii) increased cardiac biomarkers hs-cTn and NT-proBNP [1,2,3].

Hypertension is an important risk factor for the development of AF [4,5]. In particular, increased systolic blood pressure (SBP) is more associated with the presence of AF than diastolic blood pressure (DBP) [6]. Also, while the presence of HT is more common in female AF patients [7], the frequency of AF has been reported to be higher in female patients with HT [8,9]. The presence of HT in AF patients is associated with stroke, HF, major bleeding, and CV mortality [2]. The European Society of Cardiology (ESC) guideline for the diagnosis and treatment of AF recommends good blood pressure (BP) control in the association of AF and HT to reduce the AF recurrence, stroke, and cerebral hemorrhage [2]. In the presence of HT in AF patients, SBP and DBP targets are 120–129 mmHg and 70–79 mmHg, respectively. However, treatment with angiotensin-converting enzyme (ACE) inhibitors or angiotensin receptor blockers (ARBs) has been reported to protect from AF recurrence. HT, which is so closely associated with AF, is also an important part of the CHA_2_DS_2_-VA score, a thromboembolic risk marker in AF patients. Therefore, the presence of HT should be well evaluated and treated in patients with AF.

In the ESC HT treatment guideline, the most important determinant for the initiation of treatment of HT patients is a cardiovascular disease (CVD) risk of ≥10%, obtained by using the Systematic Coronary Risk Evaluation 2 (SCORE2) and Systematic Coronary Risk Evaluation 2-Older Persons (SCORE2-OP) algorithms [10,11,12]. The SCORE2 and SCORE2-OP risk prediction models incorporate traditional risk factors such as age, gender, SBP, cholesterol values, and smoking status to estimate 10-year CVD risk [9,13]. Since the occurrence of AF in HT patients is already considered HMOD, both AF and HT guidelines routinely recommend good BP control therapy [1,2]. Therefore, the use of SCORE2 and SCORE2-OP is not routinely recommended in patients with AF. For this reason and to the best of our knowledge, there were no studies on the use of SCORE2 and SCORE2-OP in the determination of long-term CVD development both in AF and after AF ablation.

Identifying reliable predictors of AF recurrence after catheter ablation holds substantial clinical importance. Numerous clinical, demographic, echocardiographic, laboratory, procedural, and post-ablation treatment-related factors have been reported to influence the likelihood of recurrence [2]. To the best of our knowledge, no data are currently available regarding the use of SCORE2 or SCORE2-OP in predicting outcomes following AF catheter ablation. Given that these algorithms incorporate multiple cardiovascular risk factors and provide an objective, quantitative risk estimation, we hypothesized that SCORE2 and SCORE2-OP may be associated with AF recurrence after catheter ablation.

In our study, we aimed to determine the effect of SCORE2 and SCORE2-OP risk scores on long-term AF recurrence and adverse CVD development after catheter ablation in paroxysmal AF patients with HT.

## 2. Methods

### 2.1. Study Population

**Figure 1:**
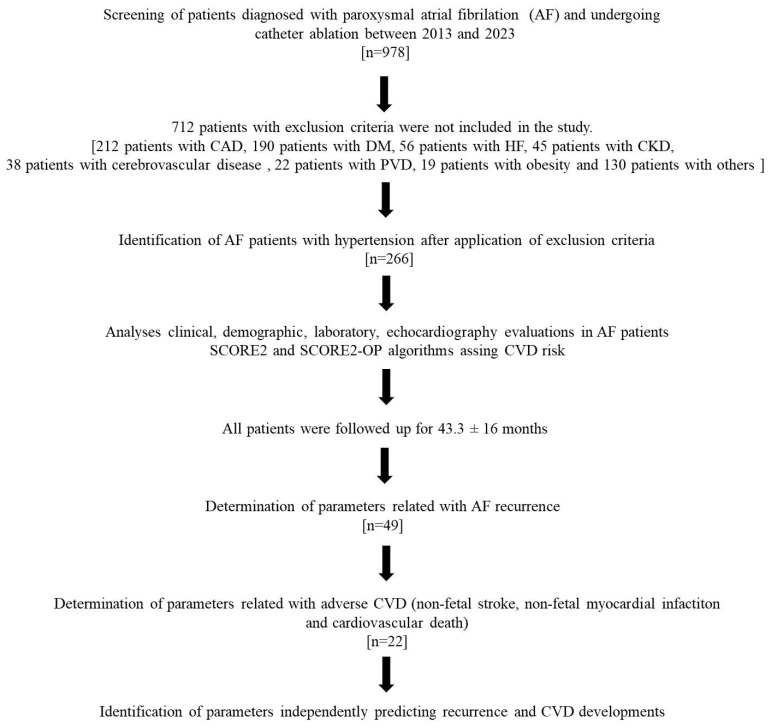
Flowchart of the study.

In our retrospective cohort study, patients who underwent AF cryoballoon ablation (CBA) with a diagnosis of paroxysmal AF in the “University of Health Sciences—Adana City Training and Research Hospital—Department of Arrhythmia Electrophysiology Laboratory” between 2013 and 2023 were screened. CBA had been performed in 978 patients diagnosed with paroxysmal AF. To determine the number of patients to be included in the study, power analysis was performed considering previous studies and their results (80% power and *p* < 0.05). After this analysis, it was found that approximately 200 patients were sufficient to be included in the study. Patients who declined participation or had coronary artery disease (CAD), heart failure (HF), diabetes mellitus (DM), an estimated glomerular filtration rate (eGFR) < 60 mL/min/1.73 m^2^ or chronic kidney disease (CKD), cerebrovascular disease (stroke or hemorrhage), familial hyperlipidemia (F-HPL), peripheral vascular disease (PVD), obesity, insufficient prognostic data during follow-up, loss to follow-up, non-CV mortality, severe valvular heart disease, hematologic disorders, active thyroid disease, chronic liver disease, pregnancy or suspected pregnancy, or cancer were excluded. A total of 712 patients who met these exclusion criteria were not included in the study. After exclusion criteria, 266 patients were included in the study. The study received approval from the Ethics Committee of our regional hospital (Adana Health Practice and Research Center; Ethics Committee No: 679). All participants were informed about the study procedures and provided written informed consent prior to enrollment. The study was conducted in accordance with the principles of the Declaration of Helsinki (1964) and its subsequent revisions. The study algorithm is shown in Figure 1.

On the day the patients were hospitalized for ablation, their anamnesis and physical examination were noted. All risk factors used in the calculation of the CHA_2_DS_2_-VA score were evaluated individually. Age, gender, HT, DM, history of CVD, and presence of vascular disease were recorded. SBP, DBP, and heart rate were obtained. The CHA_2_DS_2_-VA score was calculated with the previously mentioned parameters, as specified in the ESC 2024 AF guidelines [2] (1) C—Congestive HF [1 point], (2) H—HT [1 point], (3) A—Age ≥ 75 [2 points], (4) D—DM [1 point], S—History of Stroke [2 points], (6) V—History of Vascular Disease [1 point], (7) A—Age 65–74 [1 point]. Anti-arrhythmic, anti-coagulant, and anti-HT treatments of the patients were recorded.

Laboratory parameters of all patients on the day of CBA were recorded. Complete blood count, fasting plasma glucose, serum blood urea nitrogen (BUN), creatinine, eGFR, sodium, potassium, uric acid, total cholesterol, low-density lipoprotein (LDL) cholesterol, high-density lipoprotein (HDL) cholesterol, and triglycerides were measured. Left atrial end-diastolic (LAd) diameter and left ventricular ejection fraction (LVEF) were calculated automatically according to Simpson’s rule [14].

### 2.2. Atrial Fibrillation Diagnosis and Follow-Up

For the diagnosis of AF, 12-lead electrocardiography (ECG), 72-h Holter ECG monitoring, or the telemetry system available in the arrhythmia unit was used in accordance with the latest ESC AF guideline criteria [2]. AF was defined by the presence of: (i) irregular atrial activity and marked beat-to-beat variability in atrial cycle length (<200 ms), (ii) irregular R–R intervals, (iii) absence of distinct, repetitive P waves, (iv) fibrillatory waves of varying morphology and amplitude replacing P waves, and (v) an irregular and variable ventricular response.

The diagnosis of paroxysmal AF was confirmed by electrophysiologists who were blinded to all clinical information.

### 2.3. Atrial Fibrillation Cryoballoon Ablation Protocol

All patients underwent CBA after at least 8 h of fasting while continuing their prescribed oral anticoagulant therapy. The procedure was performed under conscious sedation (midazolam and fentanyl). Using the Seldinger technique, 8F, 6F, and 6F sheaths were inserted into the right femoral vein, right femoral artery, and left femoral vein, respectively. A diagnostic catheter was advanced into the coronary sinus via the left femoral vein. The right femoral artery sheath was used for arterial pressure monitoring and for placement of a pigtail catheter into the non-coronary cusp during transseptal puncture; blood samples for ACT measurement were also obtained from this sheath.

The 8F venous sheath in the right femoral vein was then exchanged for an 8.5F Preface long sheath. Transseptal puncture was performed under fluoroscopic and pressure guidance using a HeartSpan™ transseptal needle (Merit Medical, South Jordan, UT, USA). Following septostomy, a 28 mm cryoballoon catheter (Arctic Front Advance™, Medtronic, Minneapolis, MN, USA; or POLARx ST™, Boston Scientific, Marlborough, MA, USA) combined with an intraluminal mapping catheter (Achieve™, Medtronic, Minneapolis, MN, USA; POLARMAP™; Boston Scientific, Marlborough, MA, USA) was gently advanced into the LA through a 15-Fr steerable sheath (FlexCath™, Medtronic, Minneapolis, MN, USA; POLARSHEATH™, Boston Scientific, Marlborough, MA, USA).

Cryoablation was applied to the pulmonary veins (PVs) in a standard sequence. Each PV was first mapped using the intraluminal catheter, after which the cryoballoon was advanced and inflated. Optimal PV occlusion was confirmed via contrast injection, and the mapping catheter was positioned to record PV potentials for real-time monitoring of isolation. Cryoenergy was delivered for 180 s when the time-to-isolation (TTI) was <30 s and for 240 s when TTI was ≥30 s.

During right-sided ablation, diaphragmatic stimulation was performed using a diagnostic catheter positioned in the coronary sinus to prevent phrenic nerve palsy. Successful cryoablation was defined as complete elimination of PV potentials and demonstration of both entrance and exit block. After completion of all applications, PV isolation was reassessed after 20 min. Additional cryoenergy was administered for any PVs without persistent isolation.

### 2.4. Follow-Ups

The primary endpoint of the study was determined as the development of fatal and non-fatal CVD. All patients were followed up at 6-month intervals for at least 24 months after inclusion in the study. CVD was considered as fatal and non-fatal myocardial infarction, stroke, and sudden cardiac death due to event. The secondary endpoint of the study was determined as the development of AF recurrence. The presence of atrial tachyarrhythmia (AF or atrial flutter) of >30 s documented by 12-lead electrocardiography or Holter electrocardiography during follow-up (except for the first 3 months in AF ablation patients) was considered AF recurrence. According to the 2024 joint consensus statement on AF ablation, patients are recommended to undergo at least a 24 h continuous Holter-type rhythm monitoring every 3 months during the first year after ablation [15]. In symptomatic individuals, extended monitoring with 7-day or 14-day continuous recordings is preferable [15]. In our study, we implemented 72 h ambulatory Holter monitoring at 3-month intervals and repeated the same assessment in patients who developed symptoms. Cases with exclusion criteria during follow-up and cases with non-CV cause mortality were excluded from the study.

### 2.5. Statistical Analysis

All analyses were performed using SPSS 23.0 (SPSS for Windows 20.0, Chicago, IL, USA). Continuous variables were expressed as mean ± standard deviation, whereas categorical variables were presented as numbers and percentages. The kappa coefficient was used to assess inter- and intraobserver variability for echocardiographic parameters and risk scores. The Kolmogorov–Smirnov test was applied to evaluate the normality of distribution for continuous variables. For comparisons between two or three groups, Student’s *t*-test or one-way ANOVA was used for normally distributed variables; in the absence of normal distribution, the Mann–Whitney U test or the Kruskal–Wallis one-way ANOVA was applied, respectively. Categorical variables were compared using the chi-square test. Variables found to be significant in the univariate analyses (*p* < 0.05) were subsequently included in a multivariate logistic regression model to identify predictors of CVD and AF recurrence. In a study of this nature, multicollinearity within logistic regression analyses is an expected finding, particularly among laboratory parameters and echocardiographic measurements. However, evaluation of the Variance Inflation Factor (VIF) values indicated that no variables demonstrated elevated VIF levels during the stepwise modeling procedures. Therefore, no additional corrective processing was deemed necessary. A two-sided *p* value < 0.05 was considered statistically significant for all analyses.

## 3. Results

Of the 978 patients screened for the study, 266 (27%) patients with regular follow-up after the exclusion criteria were included in the study (144 males, 122 females, age 57.1 ± 11 years). Cohen kappa values that evaluate the interobserver variability were over 90% for all parameters (*p* < 0.001). Patients included in the study were grouped according to SCORE2 and SCORE2-OP as Group I = CVD risk < 5%, Group II = CVD risk 5 to <10%, and Group III = CVD risk ≥ 10%, and all parameters were compared. All patients were followed up for a mean of 43.3 ± 16 months for AF recurrence and adverse CVD. AF recurrence and CVD occurred in 49 (18.4%) and 22 (8.3%) patients, respectively. Patients included in the study were also grouped into those with and without AF recurrence and CVD, and parameters associated with both clinical conditions were determined.

### 3.1. Clinical, Demographic, Treatment and Follow-Up Findings of the Study Groups

Clinical, demographic, treatment, and follow-up data of the patient groups determined according to SCORE2 and SCORE2-OP are shown in Table 1. Age, smoking, SBP, CHA_2_DS_2_-VA score, and frequency of oral anti-coagulant use increased from Group I to Group III, and all parameters were significantly higher in Group III than in Group I-II. Follow-up parameters showed that the frequency of AF recurrence increased from Group I to Group III and was significantly higher in Group III patients than in Groups I-II. The incidence of CVD, non-fatal stroke, non-fatal myocardial infarction, and CV mortality was highest in Group III, and none of these conditions were observed in Group I. CVD development was found to be significantly different between all groups. Other clinical, demographic, treatment, and follow-up data were similar between all groups (Table 1).

### 3.2. Laboratory and Echocardiography Findings of the Patients Groups

Laboratory and echocardiographic data of the patient groups determined according to SCORE2 and SCORE2-OP are shown in Table 2. BUN and total cholesterol levels increased from Group I to Group III, and both parameters were significantly higher in Group III patients than in Group I. eGFR decreased from Group I to Group III and was significantly lower in Group III patients than in Group I. Other laboratory and echocardiographic data were similar between all groups (Table 2).

### 3.3. Clinical, Demographic, Treatment, Laboratory and Echocardiography Findings Associated with AF Recurrence

The patients included in the study were divided into two groups, with and without AF recurrence after follow-up, and all parameters in the study were compared between these two groups. Triglyceride levels, SCORE2 and SCORE2-OP CVD risk ≥ 10%, and Lad diameter were significantly higher in patients with AF recurrence compared to those without AF recurrence (Table 3). All other clinical, demographic, treatment, laboratory, and echocardiography data were similar in patients with and without AF recurrence.

### 3.4. Clinical, Demographic, Treatment, Laboratory, and Echocardiography Findings Associated with CVD

After follow-up, the patients were divided into two groups, with and without CVD, and all parameters in the study were compared between these two groups. Age, SBP, SCORE2 and SCORE2-OP CVD risk ≥ 10%, CHA2DS2-VA score, BUN, and uric acid levels were found to be significantly higher in patients with CVD compared to those without CVD (Table 4). All other clinical, demographic, treatment, laboratory, and echocardiography data were similar in patients with and without CVD.

### 3.5. Multivariate Logistic Regression Analysis to Identify Patients with AF Recurrence and CVD

Multivariate logistic regression analysis was performed to identify the parameters associated with AF recurrence in the univariate analysis in the whole patient group and those that independently determined patients with AF recurrence (Table 5). As a result of this analysis, the best predictors of AF recurrence were LAd diameter and the presence of SCORE2 and SCORE2-OP CVD risk ≥ 10%, respectively (Table 5). As a result of this analysis, each 1 mm LAd diameter increase and presence of CVD risk ≥ 10% increased the risk of AF recurrence by 22% and 2.45-fold, respectively.

**Figure 2:**
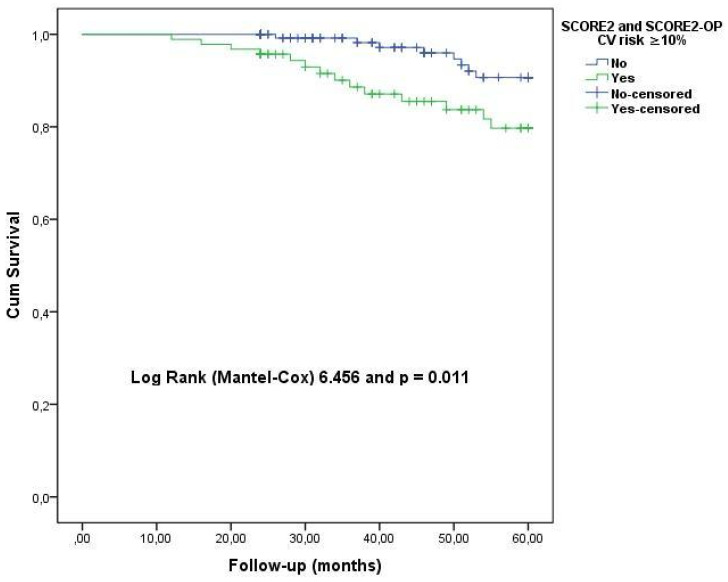
Kaplan–Meier survival curves for atrial fibrillation patients with cardiovascular (CV) disease: presence or absence of CV disease risk ≥ 10%. The curves were constructed with the Kaplan–Meier method and the *p* value was computed via log-rank test.

In the whole patient group, multivariate logistic regression analysis was performed to determine the independent predictors of CVD among the parameters associated with CVD development in the univariate analysis (Table 5). As a result of this analysis, serum uric acid level, CHA_2_DS_2_-VA score, and the presence of SCORE2 and SCORE2-OP CVD risk ≥ 10% were found to be the best predictors of CVD development (Table 5). As a result of this analysis, each 1 mg/dL uric acid increase and CHA_2_DS_2_-VA score increase by 1 unit and the presence of CVD risk ≥ 10% increased the risk of CVD by 98%, 3.26-fold, and 3.96-fold, respectively. The Kaplan–Meier survival curves in Figure 2 show that the presence of CVD risk ≥ 10% in SCORE2 and SCORE2-OP was significantly related to CVD.

## 4. Discussion

This study has several important implications, which can be summarized as follows: (1) Long-term AF recurrence rate of 18.4% in isolated HT patients undergoing catheter ablation for paroxysmal AF; (2) the best predictors of AF recurrence are LAd diameter, SCORE2 and SCORE2-OP, and the presence of CVD risk ≥ 10%, respectively; (3) each 1 mm LAd diameter increase and presence of CVD risk ≥ 10% increases the risk of AF recurrence by 22% and 2.45-fold, respectively; (4) the rate of development of long-term adverse CVD after AF ablation was 8.3%; (5) serum uric acid level, CHA_2_DS_2_-VA score and SCORE2 and SCORE2-OP CVD risk ≥ 10% are the best predictors of CVD, respectively; (6) a 1 mg/dL increase in uric acid, a 1 unit increase in CHA_2_DS_2_-VA score and CVD risk ≥ 10% increase the risk of CVD by 98%, 3.26-fold and 3.96-fold, respectively. (7) According to the results of our study, SCORE2 and SCORE2-OP algorithms performed before the procedure in isolated HT patients undergoing ablation for paroxysmal AF can be used to predict AF recurrence and CVD development.

A risk-oriented treatment approach is recommended in HT patients, taking into account comorbid CVD. The presence of certain diseases or parameters accompanying HT cases is directly associated with CVD, and in the presence of these conditions, patients are directly initiated on treatment. These can be summarized as (i) CAD, (ii) moderate to severe CKD, (iii) HMOD, (iv) DM, (v) F-HPL, (vi) cerebrovascular disease, (vii) PVD, and (viii) HF [1]. We call the presence of subclinical organ complications associated with HT and elevated BP HMOD. If left untreated, HMOD can progress from asymptomatic to symptomatic disease, ultimately resulting in overt CVD. Therefore, direct treatment is also initiated in patients with HMOD. One of the cardiac HMOD is AF.

The coexistence of HT and AF is not a coincidence. The common risk factors for these two diseases are (i) increased age, (ii) obesity, (iii) DM, (iv) smoking, (v) alcohol consumption, (vi) sedentary lifestyle, (vii) obstructive sleep apnea, (viii) CKD, (ix) family history, (x) hyperthroidism, (xi) stress, and (xii) dyslipidemia [16]. The pathophysiological mechanism of AF in these diseases is multifactorial and is associated with structural, biochemical, and electrical remodeling of the heart [16]. Prolonged HT creates an arrhythmogenic substrate in the heart by LA dilatation and fibrosis. In addition to HT, remodeling of the LA due to direct BP elevation and upregulation of the renin–angiotensin–aldosterone system and sympathetic nervous system, which are involved in the physiopathology of HT, are directly associated with the development of AF [1,2,3,4,5]. Therefore, the presence of HT should be carefully evaluated in AF patients, AF and HT should be evaluated for the presence of CVD such as concomitant CAD, and appropriate BP lowering should be initiated to prevent both disease progression and complications. We did not evaluate the frequency of AF development in HT patients in our study. Epidemiologic studies have shown that the presence of HT increases the development of AF twofold.

The ESC 2024 BP and HT treatment guideline recommends the use of SCORE2 and SCORE2-OP algorithms to determine CVD risk in HT patients without CVD and HMOD [1]. As far as we have searched in the literature, there is no data on the clinical usability of SCORE2 and SCORE2-OP algorithms in patients with AF. The most important reason for this may be that many patients with AF and HT together already have CVD or HMOD. After all, AF can be considered a variant of cardiac HMOD. We hypothesized that long-term CVD development and AF recurrence may be associated with SCORE2 and SCORE2-OP algorithms in patients with paroxysmal AF with only HT and no other HMOD and CVD. Because the risk factors used in the SCORE2 and SCORE2-OP algorithms include AF recurrence and especially AF thromboembolism risk factors [6,7,8,9,17,18,19]. Although there are no data on the use of SCORE2 and SCORE2-OP for this purpose similar to our study, most of the risk factors in these algorithms—age [17], SBP [6], total cholesterol [18], HDL cholesterol [19], and smoking status [18]—are associated with AF recurrence. The significant association between the risk factor of male gender, which is only in these algorithms, and the frequency of recurrence after AF ablation was not explained. Similarly, in patients with newly diagnosed DM, the SCORE2-Diabetes algorithm has been shown to independently determine the presence of AF as well as the development of CVD [20]. Therefore, it is very logical that the SCORE2 and SCORE2-OP algorithms were associated with AF recurrence in our study.

Although AF catheter ablation therapy reduces AF recurrence compared to medical therapy, it has not been shown to clearly reduce CV mortality and morbidity except in specific diseases (HFrEF) [2,21,22,23]. A recent meta-analysis has shown that a diagnosis-to-ablation time of <1 year provides a significant reduction in recurrence and mortality [24]. In the same study, it was reported that the development of AF recurrence was also less in patients who underwent early ablation [24]. In this meta-analysis of 23 studies and 43,711 patients, it was shown that the other 2 predictors of AF recurrence were the presence of LA diameter and HT, similar to our study [24]. Different results could have been obtained if the SCORE2 and SCORE2-OP algorithms used in HT patients were also used in this meta-analysis. Our study is not a mortality evaluation study in AF ablation and medical treatment. Long-term CV mortality after AF ablation has been shown to be 5.6%. We think that it is especially important to identify patients who may develop CVD (mortality-morbidity) in the long term after AF ablation. In this way, these patients can be followed and treated more closely. In our study, we think that SCORE2 and SCORE2-OP algorithms can be used in this regard, especially in isolated HT cases.

Current ESC hypertension guidelines consider AF as a manifestation of hypertensive-mediated organ damage (HMOD), and patients with AF are generally managed as high-risk individuals irrespective of their SCORE2 risk category. Both the AF and hypertension guidelines recommend intensive blood pressure control and comprehensive management of cardiovascular risk factors in patients with concomitant AF and hypertension, independent of SCORE classification.

Recently, Karayiannides et al. [20] demonstrated that SCORE2-Diabetes independently predicted the development of AF in patients with newly diagnosed diabetes over a 6-year follow-up period. In that study, the incidence of AF increased by 2.8-fold, 7.2-fold, and 14.6-fold among individuals classified as moderate-, high-, and very-high-risk according to SCORE2-Diabetes, respectively. Our study differs from that work in that patients with diabetes were excluded. We found that isolated hypertensive patients classified as high-risk according to SCORE2 or SCORE2-OP exhibited a 2.45-fold higher likelihood of paroxysmal AF recurrence following CBA.

Although hypertension contributes to both the development of AF and the risk of recurrence after ablation, early identification of high-risk individuals using an objective and multifactorial tool such as SCORE2 and SCORE2-OP may hold meaningful clinical value. Patients with elevated SCORE2 and SCORE2-OP scores may warrant closer follow-up for AF recurrence, more intensive risk-factor modification, prolonged antiarrhythmic therapy, and enhanced patient counseling to minimize recurrence risk. Nevertheless, our findings require confirmation in larger, prospective studies.

In our analysis, SCORE2 and SCORE2-OP, the CHA_2_DS_2_-VA score, and left atrial dimension (LAd) were all associated with AF recurrence. LAd remains one of the most important and well-established predictors of recurrence [2,15,17,24,25]. However, in isolated hypertensive patients—particularly those with normal LAd—SCORE2 and SCORE2-OP assessment may provide additional prognostic information.

### Limitations

Our study was a single-center, retrospective study with a limited number of cases in a single ethnic region. Therefore, prospective, multicenter studies including more cases and cases of different ethnic origins are needed. In our study, patients with moderate to severe CKD, known CVD, DM, F-HPL, HF, obesity, cerebrovascular disease, and PVD, which are important in the treatment and prognosis of AF + HT patients, were included as exclusion criteria. Therefore, our data cannot be used for all AF patients. Age is a major risk factor for cardiovascular disease and the strongest predictor of stroke in the presence of AF. In our study, advanced age was not used as an exclusion criterion. However, a study restricted to individuals aged 40–65 years may have provided a more meaningful evaluation of the predictive performance of SCORE2 and SCORE2-OP for determining AF recurrence after CBA in patients with paroxysmal AF. Carotid-femoral Doppler ultrasound, pulse wave velocity evaluation, urinary albumin/creatinine measurement, and NT-proBNP examinations, which are necessary for the detection of HMOD in HT patients, were not performed. These evaluations would have yielded more significant results. In patients with HT, appropriate BP control has been shown to reduce both AF development and AF recurrence [15]. Unfortunately, we did not make this examination in our patients. SCORE2 and SCORE2-OP estimate the 10-year risk of cardiovascular events; however, the follow-up period in our study was a minimum of 24 months, with a mean duration of 43.3 ± 16 months, which does not approach a full 10-year interval. While this follow-up period is adequate for evaluating AF recurrence, it is insufficient for reliably assessing long-term cardiovascular outcomes.

## 5. Conclusions

According to the results of our study, SCORE2 and SCORE2-OP algorithms performed before the procedure in isolated HT patients undergoing CBA for paroxysmal AF predict AF recurrence and CVD development. Therefore, these algorithms, which are used to determine CVD risk and initiate medical treatment in patients with HT, can also be used to determine the development of CVD and AF recurrence in hypertensive AF patients. Furthermore, in isolated hypertensive patients undergoing AF ablation, those with a SCORE2 (or SCORE2-OP)–estimated cardiovascular risk ≥ 10% may require closer follow-up and more intensive management to reduce the likelihood of recurrent AF. The SCORE2 and SCORE2-OP algorithms were evaluated only in isolated hypertensive patients, and further studies are needed to determine their applicability in the broader AF population. However, prospective, multicenter studies with more patients should be conducted in order to make more robust and clear recommendations on this subject.

## Figures and Tables

**Table 1:** Clinical, demographic, treatment, and follow-up findings of the patients group.

Variable	Group I *n* = 67	Group II *n* = 106	Group III *n* = 93	*p*
Age (year)	46.1 ± 7.4 ^α,β^	56.7 ± 8.1 ^¥^	67.2 ± 11	**<0.001**
Gender (Male), *n*	39 (58%)	59 (56%)	46 (50%)	0.505
Current smoking, *n* (%)	4 (6%) ^a^	16 (15%) ^a^	36 (39%) ^b^	**0.001**
Systolic blood pressure (mmHg)	137 ± 16 ^α^	139 ± 15	143 ± 14	**0.037**
Diastolic blood pressure (mmHg)	81 ± 13	81 ± 11	81 ± 10	0.998
Heart rate (beat/min)	83 ± 13	82 ± 14	82 ± 112	0.898
CHA_2_DS_2_-VA score	1.0 ± 0.0 ^α,β^	1.15 ± 0.36 ^¥^	1.74 ± 0.71	**<0.001**
Propafenone use, *n* (%)	17 (25%)	22 (21%)	22 (24%)	0.764
Flecainide use, *n* (%)	20 (30%)	18 (17%)	24 (26%)	0.143
Amiodarone use, *n* (%)	15 (23%)	38 (36%)	28 (30%)	0.380
Beta blocker or CCB use, *n* (%)	61 (91%)	91 (87%)	86 (93%)	0.837
ACE inhibitor or ARB use, *n* (%)	36 (54%)	58 (55%)	53 (57%)	0.674
Oral anti-coagulant use, *n* (%)	23 (34%) ^a^	50 (47%) ^a^	52 (56%) ^b^	**0.009**
Atrial fibrillation recurrence, *n* (%)	9 (13%) ^a^	17 (16%) ^a^	23 (25%) ^b^	**0.035**
Cardiovascular disease, *n* (%)	0 (0%) ^a^	8 (8%) ^b^	14 (15%) ^b^	**0.001**
Non-fatal stroke, *n* (%)	0 (0%) ^a^	6 (6%) ^a^	8 (9%) ^b^	**0.011**
Non-fatal myocardial infarction, *n* (%)	0 (0%)	2 (2%)	2 (2%)	0.294
Cardiovascular mortality, *n* (%)	0 (0%) ^a^	0 (0%) ^a^	4 (4%) ^b^	**0.014**

The values were shown as mean ± standard deviation or *n* (%), ACE: Angiotensin converting enzyme, ARB: Angiotensin receptor blockers, CCB: Calcium canal blocker, statistically significant *p* values were shown in bold. Group I = cardiovascular disease risk < 5% in SCORE2 and SCORE2-OP. Group II = cardiovascular disease risk 5 to <10% in SCORE2 and SCORE2-OP. Group III = cardiovascular disease risk ≥ 10% in SCORE2 and SCORE2-OP. ^α^ = the significant association between Group I and Group II (*p* < 0.05). ^β^ = the significant association between Group I and Group III *p* < 0.05). ^¥^ = the significant association between Group II and Group III (*p* < 0.05). ^a^: no significant association between groups (*p* > 0.05) ^b^: significant association between groups (*p* < 0.05).

**Table 2:** Laboratory and echocardiography findings of the patients’ groups.

Variables	Group I *n* = 67	Group II *n* = 106	Group III *n* = 93	*p*
White blood cell (10^3^/µL)	7.32 ± 2.4	7.51 ± 2.3	7.39 ± 1.9	0.848
Platelet count (10^3^/µL)	266 ± 71	250 ± 75	251 ± 77	0.481
Hemoglobin (g/dL)	13.4 ± 1.5	13.6 ± 1.4	13.5 ± 1.5	0.703
Fasting plasma glucose (mg/dL)	102 ± 19	103 ± 17	105 ± 21	0.353
Creatinine (mg/dL)	0.70 ± 0.16	0.78 ± 0.48	0.76 ± 0.32	0.368
Blood urea nitrogen (mg/dL)	24.8 ± 6.7 ^α^	28.4 ± 7.8	29.6 ± 11	**0.004**
eGFR (mL/min/1.73 m^2^)	103 ± 18 ^α^	98 ± 14	94 ± 16	**0.003**
Uric acid (mg/dL)	4.57 ± 1.4	4.86 ± 1.3	5.13 ± 1.8	0.069
Sodium (mmol/L)	140 ± 2.4	140 ± 2.4	139 ± 2.9	0.123
Potassium (mmol/L)	4.28 ± 0.46	4.22 ± 0.42	4.25 ± 0.37	0.644
Total cholesterol (mg/dL)	185 ± 43 ^α^	197 ± 51	204 ± 49	**0.042**
HDL cholesterol (mg/dL)	51.3 ± 13	49.6 ± 14	47.2 ± 9.6	0.109
LDL cholesterol (mg/dL)	124 ± 32	133 ± 34	132 ± 34	0.161
Triglycerides (mg/dL)	166 ± 81	174 ± 121	191 ± 143	0.382
LVEF (%)	59.5 ± 3.1	59.3 ± 3.7	58.8 ± 4.2	0.399
LA dimension (mm)	37.8 ± 4.3	36.9 ± 4.6	37.9 ± 4.4	0.230

The values were shown as mean ± standard deviation or *n* (%), eGFR: Estimated glomerular filtration rate, HDL: High-density lipoprotein, LA: Left atrium, LDL: Low-density lipoprotein, LVEF: Left ventricular ejection fraction, statistically significant *p* values were shown in bold. Group I = cardiovascular disease risk < 5% in SCORE2 and SCORE2-OP. Group II = cardiovascular disease risk 5 to <10% in SCORE2 and SCORE2-OP. Group III = cardiovascular disease risk ≥ 10% in SCORE2 and SCORE2-OP. ^α^ = the significant association between Group I and Group II (*p* < 0.05).

**Table 3:** Clinical, demographic, treatment and laboratory findings associated with AF recurrence.

Variables	AF Recurrence (+) *n* = 49	AF Recurrence (−) *n* = 217	*p*
Triglycerides (mg/dL)	183 ± 87	153 ± 85	**0.036**
SCORE2(OP) CVD risk, <5%-5 to <10%-≥10%, *n*	9-17-23	58-89-70	**0.035**
Left atrium dimension (mm)	40.4 ± 5.6	36.8 ± 3.8	**<0.001**

The values were shown as mean ± standard deviation or *n* (%), AF: Atrial fibrillation, SCORE(OP): SCORE2 or SCORE2-OP. Statistically significant *p* values were shown in bold.

**Table 4:** Clinical, demographic, treatment, and laboratory findings associated with adverse CVD.

Variables	Adverse CVD (+) *n* = 22	Adverse CVD (−) *n* = 244	*p*
Age (year)	63.2 ± 12	57.2 ± 11	**0.018**
Systolic blood pressure (mmHg)	140 ± 15	135 ± 15	**0.048**
SCORE2(OP) CVD risk, <5%-5 to <10%-≥10%, *n*	0-8-14	67-98-79	**0.001**
CHA_2_DS_2_-VA score	1.77 ± 0.81	1.28 ± 0.53	**0.010**
Blood urea nitrogen (mg/dL)	31.4 ± 12	27.6 ± 11	**0.028**
Uric acid (mg/dL)	6.33 ± 2.6	4.75 ± 1.3	**0.009**

The values were shown as mean ± standard deviation or *n* (%), CVD: Cardiovascular disease, SCORE(OP): SCORE2 or SCORE2-OP. Statistically significant *p* values were shown in bold.

**Table 5:** Multivariate logistic regression analysis to identify patients with AF recurrence and CVD.

**AF Recurrence**	**Odds Ratio**	**95% CI**	***p***
SCORE2(OP) CVD risk (presence of ≥10%)	2.448	1.324–4.126	**0.005**
Left atrium dimension (each 1 mm)	1.217	1.124–1.319	**<0.001**
**CVD**	**Odds Ratio**	**95% CI**	***p***
SCORE2(OP) (presence of CVD risk ≥ 10%)	3.960	1.098–8.408	**<0.001**
CHA_2_DS_2_-VA score (each 1)	3.257	1.067–7.318	**<0.001**
Uric acid (each 1 mg/dL)	1.976	1.359–2.873	**0.001**

Statistically significant *p* values were shown in bold. AF: Atrial fibrillation, CVD: Cardiovascular disease, SCORE(OP): SCORE2 or SCORE2-OP.

## Data Availability

The data supporting the findings of this study are not publicly available due to ethical consideration and the protection of patient privacy.
